# The effects of psychotherapeutic approaches in children and adolescents with intellectual disabilities and psychological disorders: a meta-analysis

**DOI:** 10.1007/s00787-026-03018-2

**Published:** 2026-03-28

**Authors:** Amanda Amazu, Johannes Michalak, Aleksandra Kaurin, Bodo Przibilla, Vinzenz Gardenier, Vera Swenshon, Johannes Graser

**Affiliations:** 1 Department of Psychology and Psychotherapy, Witten/Herdecke University, Alfred‑Herrhausen‑Straße 50, D‑58448 Witten, Germany; 2 University of Wuppertal, Wuppertal, Germany

**Keywords:** Children, Intellectual disability, Psychotherapeutic intervention, Behavioural disorders, Parenting stress

## Abstract

Children and adolescents with intellectual disabilities (ID) are two to three times more likely to develop mental disorders, with prevalence rates up to 40%. While meta-analyses demonstrate the efficacy of psychotherapeutic interventions in adults, the evidence base for younger populations remains limited. A systematic literature search was conducted across seven databases following PRISMA guidelines. Studies were included if they examined psychotherapeutic interventions for participants aged 3–21 years with borderline intellectual functioning to severe ID (IQ 20–84) and reported quantitative outcomes. The analysis of 16 studies showed a small but significant effect on behavioural disorders across 14 controlled trials (*SMD* = -0.26, *p* = .03; *I²* = 30%). Severity of ID did not moderate outcomes, but individually delivered interventions were more effective (*SMD* = -0.46) than group-based formats. No effect was found for parental stress (*SMD* = -0.10, *p* = .27; *I²* = 0%). Evidence for internalising disorders came only from a few uncontrolled studies, suggesting large but unreliable effects. Psychotherapeutic interventions thus offered modest benefits for behavioural problems in youth with ID, while clear gaps remain for parental stress and internalizing conditions.

## Public health significance

These findings indicate that psychotherapeutic interventions for children and adolescents with ID may produce modest improvements in behavioural disorders. While preliminary evidence from a limited number of uncontrolled studies suggests potentially promising effects for internalising symptoms such as anxiety and depression, this area remains profoundly neglected in controlled research, leaving clinical practice without an evidence-based foundation. This differential efficacy highlights the need for tailored, evidence-based mental health strategies and substantially more high-quality research to better support this vulnerable population’s diverse therapeutic needs.

## Introduction

Intellectual disability (ID) – termed *intellectual developmental disorder (IDD)* in the Diagnostic and Statistical Manual of Mental Disorders, Fifth Edition, Text Revision (DSM-5-TR; American Psychiatric Association, [3]) – encompasses substantial limitations in both cognitive functioning and adaptive behaviour that emerge during developmental periods prior to adulthood. These limitations significantly impact an individual’s conceptual understanding, social interactions, and practical daily living skills. Similarly, the World Health Organization [57] characterises ID as a condition marked by impaired cognitive development and diminished capacity to adapt to environmental demands and social expectations. Both definitions emphasise that onset occurs during the developmental period before the age of 18 years. The American Association on Intellectual and Developmental Disabilities [4] further clarifies that these functional limitations necessitate individualised support systems across various life domains to facilitate participation in community settings and activities.

Various epidemiological studies have consistently shown that people with ID have a two to three times higher risk of developing mental disorders compared to the general population [10, 13]. This increased prevalence can be attributed to a variety of biological, psychological and social risk factors, such as genetic predispositions, difficulties in emotion regulation, limited coping strategies, social exclusion and trauma [58]. However, the precise prevalence rates vary depending on the specific study design, diagnostic criteria and the sample analysed [10]. Focusing on child and adolescent mental health reveals that children and adolescents with ID are at increased risk for a broad range of comorbid mental disorders [13, 25]. Epidemiological studies indicate that up to 40% of this population are affected, exceeding prevalence rates observed in the general population [33]. More recent epidemiological data corroborate these findings, with large population-based and clinical studies reporting prevalence estimates ranging from approximately 30% to 50% (Platt et al., 2019; Totsika et al., 2022; Tural Hesapcioglu et al., 2019). Within this group, behavioural disorders, anxiety disorders, and depressive disorders represent the most frequently diagnosed categories, with reported prevalence rates of approximately 30–40% for behavioural disorders, 20–30% for anxiety disorders, and around 10–20% for depressive disorders [8, 55]. Beyond elevated prevalence rates, the clinical presentation of psychiatric disorders in children and adolescents with ID often differs in quality and expression from that observed in typically developing populations. Behavioural disorders, most commonly attention-deficit/hyperactivity disorder, oppositional defiant disorder, and conduct disorder, frequently present through impulsive, aggressive, or dysregulated behaviours, including self-injurious tendencies, which may obscure underlying emotional distress and complicate differential diagnosis [33]. Anxiety disorders often manifest through withdrawal behaviour, heightened fear responses, and somatic complaints such as sleep disturbances or abdominal pain [13]. Depressive symptoms in this population are frequently characterised by reduced motivation, social withdrawal, irritability, and persistent low mood rather than overt verbal expressions of sadness [25]. In addition, internalising conditions such as obsessive–compulsive disorder may occur in children and adolescents with ID, often presenting in atypical or behaviourally mediated forms. Rather than being expressed through clearly verbalised obsessions or distress, symptoms may manifest through repetitive behaviours, rigidity, or self-injurious acts, complicating differentiation from other behavioural or developmental phenomena [55]. Such atypical symptom expression, along with stress-related socio-developmental regression, poses substantial challenges for accurate psychiatric diagnosis in this population [48]. In general, similar therapeutic approaches are available for the psychotherapeutic treatment of children and adolescents with ID and comorbid mental disorders as for patients without disabilities but require adaptations to the cognitive and communicative abilities of the patients [56]. Cognitive behavioural therapy (CBT) aims to reduce anxiety, compulsions or depressive symptoms through psychoeducation, exposure and restructuring of dysfunctional thoughts (Fynn. et al., 2023; [52]). Behavioural therapy techniques, play therapy and parent-child interaction training are particularly suitable for behavioural problems, attention disorders and for younger or severely impaired patients [5]. In addition, psychodynamic, gestalt therapy or systemic approaches are occasionally applied [21].

Despite the need for effective psychotherapeutic interventions for children and adolescents with ID and co-morbid mental health conditions, treatment options remain limited [15]. Psychotherapists often lack specific training for treating people with ID [12] and report feeling insufficiently qualified to work with this client group. Additionally, diagnosis and setting realistic treatment goals are challenging due to the specific cognitive and behavioural patterns of this patient group [42]. Structural barriers impede access to psychotherapeutic services, and treatment motivation frequently originates from the patient’s environment rather than the individual themselves, potentially creating goal conflicts [37].

In recent years, several systematic reviews and meta-analyses have investigated the efficacy of psychotherapeutic interventions for adults with ID. A meta-analysis by Vereenooghe and Langdon [53] showed that cognitive behavioural therapy is effective for the treatment of depression in adults with ID (*g* = 0.74). More recently, Tapp et al., [49] conducted an updated meta-analysis of psychological therapies for people with ID. Their findings showed a small but significant overall effect size (*g* = 0.43, 95% CI [0.20, 0.67]) across various mental health problems. For specific conditions, psychological therapy showed moderate efficacy for anger (*g* = 0.60), while treatments for depression and anxiety yielded smaller, non-significant effects (*g* = 0.38).

A meta-analysis conducted by Graser et al., [18] underlines the efficacy of cognitive behavioural therapy (CBT) in the treatment of depression and anger/aggression in adults with intellectual impairment, with significant effects with an effect size of *d* = 0.65 observed in both domains. Furthermore, in their meta-analysis Nicoll et al., [34] found that cognitive behavioural therapy (CBT) can be highly effective for adults with ID and comorbid psychopathological symptoms, more specifically for the reduction of anger and aggressive behaviour. While the efficacy of psychotherapy for adults with ID has been substantiated in several meta-analyses, the empirical foundation for comparable interventions in younger populations remains considerably more limited. While a number of meta-analyses have already presented evidence for the efficacy of psychotherapy for adults with ID, the evidence base for children and adolescents is much more limited.

Up until now, the only meta-analysis specifically addressing psychotherapeutic interventions for children and adolescents with intellectual and developmental disabilities has been conducted by Lory et al., [30]. The authors examined the efficacy of interventions targeting children from preschool to elementary school age in inclusive educational settings. A total of 15 studies were included in the analysis, highlighting moderate to large effects across behavioural and emotional outcome domains. They found a large effect size (*d/g* = 0.94) for interventions targeting challenging behaviours such as aggression and self-injurious behaviour. However, while individual studies exist, there are currently no meta-analyses addressing the treatment of other comorbid mental disorders such as anxiety or depression with psychotherapy in children and adolescents with ID. Meta-analyses examining psychotherapeutic interventions for children with ID remain notably limited in scope and comprehensiveness. While Lory et al., [30] evaluated intervention efficacy for challenging behaviours in elementary-aged children, Vereenooghe et al., [54] reviewed treatments for children with severe and profound ID concluding that the evidence base remains extremely limited and insufficient to inform clinical guidelines, no systematic synthesis has addressed the broader spectrum of common mental health conditions in this population.

This meta-analysis examines available therapeutic interventions for prevalent psychological disorders in children and adolescents with ID and assesses their efficacy to line out the current intervention landscape. By quantifying intervention effects in this underserved population, this meta-analysis will provide clinicians with an empirical foundation for treatment selection and inform service development priorities. Furthermore, systematic identification of methodological limitations in the current evidence base will generate specific recommendations for future research, potentially catalysing methodological improvements in this challenging but essential area of clinical research.

## Methods

This meta-analysis was registered in the International Prospective Register of Systematic Reviews (PROSPERO; registration number CRD42024479583). Further, the present meta-analysis was conducted in accordance with the guidelines of the Preferred Reporting Items for Systematic Reviews and Meta-Analyses (PRISMA). An ethics approval was not required since only data from published studies were analysed. This meta-analysis included studies focusing on children and adolescents diagnosed with borderline intellectual functioning and ID ranging from mild to severe (IQ 20–69). The age range of the participants was purposefully set at 2 to 21 years to enable a comprehensive representation of the developmental psychological stages from infancy to young adulthood. This range aligns with educational service eligibility criteria for individuals with ID in many countries (Individuals with Disabilities Education Act in the US; [16]). Early childhood (ages 2–5) represents a critical period for developmental intervention and early identification of comorbid conditions [19]. Middle childhood and adolescence (ages 6–17) correspond to periods of increasing social demands and heightened risk for internalising disorders [13]. The upper age limit of 21 years was chosen to reflect the frequently delayed developmental trajectory observed in individuals with ID and to allow the inclusion of transition-focused interventions aimed at bridging pediatric and adult mental health services [17]. This decision also accounts for the structure of age-specific psychotherapeutic care systems, in which transition planning is typically embedded in late adolescence or early adulthood.

Furthermore, studies were included that investigated the effects of a structured psychotherapeutic intervention. Here, both group-based and individual therapy approaches as well as parent training programmes were involved, provided that these were led by qualified professionals. These interventions were based on established psychotherapeutic frameworks, including cognitive-behavioural, psychodynamic, analytical or systemic approaches. A further criterion was the use of standardised and validated psychometric instruments to measure therapy outcomes, specifically regarding symptoms of ADHD, anxiety, depression, and anger. To ensure comprehensive coverage of available evidence, randomised controlled trials (RCTs), controlled studies, and uncontrolled studies that included pre- and post-intervention measurements were included. We required that the studies reported means and standard deviations to allow us to calculate effect sizes for consistent and comparable analyses across studies. Due to the language capabilities of the review team, only studies in English and German were included. Studies with adult participants (aged 21 and over) and those that included individuals with average intellectual performance were excluded. Studies were also excluded if they did not provide sufficient statistical evidence or if they did not provide such evidence when requested by the authors.

The systematic literature review was conducted in November and December 2023, utilising databases including PsycINFO, PubMed, Cochrane Library, Google Scholar, Web of Science, EBSCOhost, and Scopus. An updated search was conducted in July 2025 to account for recent database updates. The search algorithm contained a detailed specification of search terms and Boolean operators, adapted for each individual database to maximise the precision and relevance of the results. The search terms consisted of a combination of the target population, the type of intervention, and the study design. The search term applied was:

TITLE-ABS-KEY (psychotherapy OR “cognit* behav* therap*” OR CBT OR “cbt” OR “behavior* therapy” OR “cognitive* therapy” OR “dialectical behavior* therapy” OR “acceptance commitment therapy” OR “solution focused therapy” OR mindfulness OR psychoeducation OR “social skills training” OR “anger management” OR “emotion regulation” OR “family systems therapy” OR “interpersonal therapy” OR “psychodynamic* therapy” OR humanistic OR experiential OR “functional analytic psychotherapy” OR “habits therapy” OR “relational frame therapy” OR counseling) AND (“intellect* disability” OR “learning disability” OR “development* disability” OR “ment* retard*”) AND (child* OR adolescent* OR teen* OR youth* OR “adolescen*”) AND (“mental health” OR “mental disorders” OR psychiatr* OR psycholog* OR “mental illness” OR anxiety OR depression OR adhd).

Particular attention was paid to adjusting the search string made for PubMed using mesh terms to optimise the accuracy of the search within the rather medically oriented database. In addition, complementary strategies such as snowballing, hand searching and expert queries were used to ensure that studies that may not have been listed in the main databases were found.

The data collection in this meta-analysis involved the extraction of relevant data for each included study using a standardised data collection table. These data included key study characteristics such as first author, year of publication, study location, study design (randomised controlled trials, controlled or uncontrolled studies) and participant characteristics (age range, gender distribution). Attention was paid to intervention characteristics (type - individual, group, parent training; duration; frequency; theoretical approach - cognitive-behavioural, psychodynamic/analytic, systemic) and outcome measures.

Outcome measures included:

1. **Behavioural Disorders**: Changes in externalising symptoms such as aggression, hyperactivity, impulsivity, and self-injurious behaviours were evaluated. Studies typically employed standardised behavioural rating scales, including, but not limited to, the Child Behavior Checklist (CBCL; [1]), the Aberrant Behavior Checklist (ABC; [2]), or the Developmental Behaviour Checklist (DBC; [14]).
2. **Anxiety**: Reductions in anxiety symptoms were captured using various validated anxiety scales. Frequently used measures included both general anxiety questionnaires (e.g., Spence Children’s Anxiety Scale – Parent Version [SCAS-P]; [45]) and disorder-specific scales adapted for individuals with ID (e.g., Screen for Child Anxiety Related Disorders – Parent Version [SCARED-P]; [6]).
3. **Depression**: Improvements in depressive symptoms were assessed primarily through instruments that screen broadly for emotional difficulties, including depressive symptoms such as sadness, loneliness, or feelings of worthlessness. Commonly used measures included general screening instruments like the Youth Self Report (YSR; [1]).
4. **Parenting Stress**: Changes in parental stress levels were included due to their relevance for intervention implementation and overall family well-being. Parental stress directly influences caregiving capacity, parent-child interactions, and long-term treatment outcomes. Frequently employed instruments included the Parenting Stress Index (PSI; Abidin, 1995), which assesses various dimensions of parental stress, including perceived parental distress, difficult parent-child interactions, and overall perceptions of child difficulty.

## Risk-of-bias rating

The methodological quality of the included studies was assessed using two complementary instruments: the Cochrane Collaboration’s Risk of Bias Tool 2 (RoB 2; [46]) for randomised controlled trials and controlled trials, and the Risk of Bias in Non-randomised Studies of Interventions (ROBINS-I; [46]) for non-randomised studies. Both tools assess methodological quality on a domain-based level rather than through summary scores. The Cochrane Risk of Bias Tool 2 (RoB 2) classifies randomised studies as having “low risk”, “some concerns” or “high risk” of bias based on five predefined domains: (1) bias arising from the randomisation process, (2) deviations from intended interventions, (3) missing outcome data, (4) measurement of the outcome, and (5) selection of the reported result.

The ROBINS-I tool, applied to non-randomised studies, evaluates seven domains, including confounding, selection of participants, classification of interventions, deviations from intended interventions, missing data, outcome measurement, and selection of reported results. Each domain is rated individually and contributes to an overall risk-of-bias judgment: “low,” “moderate,” “serious,” or “critical” risk of bias.

All assessments were independently conducted by two trained research assistants with a bachelor degree in psychology. Raters followed the decision algorithms provided in the respective RoB assessment tools. Discrepancies were resolved through structured consensus discussions or, if needed, by a final adjudication from the first author. The complete results of the risk-of-bias ratings are summarised in Table 1. For transparency, it should be noted that in five trials, only a single rater performed the RoB-2 assessment, because the second trained research assistant was no longer available. To mitigate potential bias, these single-rated assessments were subsequently reviewed by the first author using the same decision algorithms. Two studies [41, 47], identified after the initial rating phase in 2023, were assessed using the same risk-of-bias tools and decision, with final ratings reviewed by the first author.

**Table 1:** Characteristics of included studies

Study	Study design	Participants/ID severity	Intervention	Measures	Results	Ratings
Bagner and Eyberg, [5]	Randomised Controlled Trial (RCT), pre-post, 4-month follow-up	*N* = 30 IG: *n* = 15 CG: *n* = 15 Age range: 3–6 years 77% boys; age: *M* = 54.13 months (*SD* = 10.15) ID: mild to moderate (60% mild [IQ 55–75], 40% moderate)	Parent-Child Interaction Therapy (PCIT), standard protocol without modification; Conducted by clinical psychology doctoral students trained in PCIT Treatment fidelity monitored through videotape review and weekly supervision,	Child Behavior Checklist (CBCL)*; Eyberg Child Behavior Inventory (ECBI)*	Compared to the waitlist control group, the immediate treatment group showed greater child compliance, fewer disruptive behaviours (ECBI Intensity and Problem Scales), more positive parenting and effective commands, and lower parenting stress (PSI-SF Difficult Child subscale). No significant differences were found on CBCL Externalising or Total scales.	High Risk (ROB 2)
Blakeley-Smith et al. (2020)	Non-controlled pilot and feasibility study, pre-post Pilot and feasibility study, pre-post treatment	*N* = 23 (19 completed); Adolescents with ASD and ID; 91% male; Age 12–19 years (*M* = 15.9 years) ID: borderline to moderate (IQ M = 58.3, range 40–79)	Adapted cognitive behavioural therapy (CBT), Facing Your Fears” program; 14 weekly 90-minute sessions; conducted by licensed psychologists	Anxiety Depression and Mood Scale (ADAMS)*; Child Anxiety Related Disorders-Parent Report (SCARED-P)*	Significant reductions in anxiety symptoms were observed post-intervention on both ADAMS and SCARED-P	Critical Risk (ROBINS-I)
Blankestein et al., [7]	Controlled Trial (CT), comparative design (MST-ID vs. MST), 6-month follow-up	*N* = 128 (MST-ID:55, Standard MST: 73) IG: 55 CG: 73 Age range: Mean age: MST-ID = 15.2 years, Standard MST = 14.9 years. *SD*: MST-ID = 1.73 years, Standard MST = 1.38 years. ID: borderline to mild (IQ 50–85)	Multisystemic Therapy for Intellectual Disabilities (MST-ID), adapted for adolescents with ID and antisocial/delinquent behaviour; Duration 3–5 months	Child Behaviour Checklist (CBCL)* Youth Self Report (YSR)* Opvoedingsbelasting Vragenlijst for parenting stress (OBVL)*	MST-ID resulted in reduced police contact and rule-breaking behaviour lasting up to 6 months post-treatment. MST-ID showed improvements in parenting skills, family relations, social support	Critical Risk (ROBINS-I)
Hand et al., [20]	RCT, pre-post (8 weeks intervention)	*N* = 29 (parents) IG: *n* = 16 CG: *n* = 13 Age range of children: 6–12 years ID: mild (IQ not reported)	Parents Plus Children’s Programme (PPCP) a video-modelled, group-based parent training program, 2.5 h weekly for eight sessions, delivered by speech therapists	Strengths and Difficulties Questionnaire (SDQ)*, Parent Stress Index (PSI)*, Kansas Parent Satisfaction Scale (KPS)*;	The treatment group showed significant improvements: child behaviour problems decreased, shifting from borderline to normal; parental stress declined and parental satisfaction increased from low to above average.	High Risk (ROB 2)
Haydicky et al., [23]	Controlled Trial (CT), waitlist control, pre-post	*N* = 60 IG: *n* = 28 boys; age 12–18 CG: *n* = 32 boys; age 12–18 ID: learning disabilities (IQ not reported)	Integra Mindfulness Martial Arts (MMA) - manualised 20-week group program incorporating mindfulness meditation, CBT, behavior modification, mixed martial arts; conducted by trained facilitators	BRIEF (Behavior Rating Inventory of Executive Function)*; CBCL (Child Behavior Checklist)*; YSR (Youth Self Report)*	Significant improvements in parent-rated externalising behaviour, oppositional defiant problems, and conduct problems for ADHD subgroup; Notable improvements in social problems and monitoring skills for those with hyperactive/impulsive symptoms; Decreased anxiety reported by participants in the anxiety subgroup.	Critical Risk (ROBINS-I)
Ingersoll et al., [24]	Randomised Controlled Trial (RCT), pilot feasibility study, pre-post design	*N* = 20 IG: *n* = 10; 9 males/1 female; age: *M* = 16.26 (SD = 3.15) CG: *n* = 9; 7 males/2 females; age: *M* = 16.90 (SD = 2.53) ID: severe (IQ not reported)	Reciprocal Imitation Training (RIT), 2 × 10-minute sessions per day, 3 days per week for 5 months; conducted by trained teachers	Matson Evaluation of Social Skills for Individuals with Severe Retardation (MESSIER; interview)*; Aberrant Behaviour Checklist-Residential (ABC-R; checklist)*; Unstructured Imitation Assessment (UIA)*; Motor Imitation Scale (MIS)*	Moderate to large effects on social functioning (MESSIER) and challenging behaviour (ABC-R) in favor of the treatment group Mixed findings for imitation skills (UIA, MIS)	High Risk (ROB 2)
Kostulski et al., [26]	Randomised Controlled Trial (RCT), waitlist control group, pre-post design	*N* = 42 Age: *M* = 11.19 years (*SD* = 2.87), Age range 6–16 years Gender ratio: 36 male (86%) ID: mild to moderate (26% mild, 74% moderate; IQ not reported)	Parent Management Training (PMT) group program 10 sessions of 90 min over 6 months, two trainers per group, parents of up to 8 children 10 standard modules, 20 optional modules based on specific problems	Developmental Behaviour Checklist (DBC; primary outcome)*, Impact on Family Scale (FaBel)*, Questionnaire for Positive and Negative Parent Management Practices (FPNE)*	Significant improvements in behavioural and emotional problems, reduced family burden, and enhanced positive parenting in the PMT group compared to controls.	Some Concerns (ROB 2)
McIntyre (2008)	Randomised Controlled Trial (RCT), pre-post, (14–16 weeks between measurements)	*N =* 44 (families) IG: *n* = 21 CG: *n* = 23 Age range children: 2–5 years Age: *M* = 4.11 years (*SD* = 1.0); ID: developmental delays (VABS 45–85; IQ not reported)	Incredible Years Parent Training Program with developmental delay modifications, 12 weekly group sessions × 2.5 h, delivered by specialist in mental retardation	Family Impact Questionnaire (FIQ)*, Child Behavior Checklist (CBCL)*, Behavioral observation (Parent-Child Interaction)	Compared to usual care control group, intervention group showed significant improvements in: CBCL Total Problems, Internalizing, negative parent-child interactions.	Some Concerns (ROB2)
Ooms-Evers et al. (2021)	Uncontrolled pilot study, pre- and post-measurement, 3 months follow-up	*N* = 33 19 girls (57.6%), 14 boys (42.4%) Age: *M* = 13.79 years (*SD* = 2.96), 6–17 years ID: borderline to mild (IQ M = 77.85, SD = 5.57)	Trauma exposure, EMDR, Daily 2 individual sessions of 60 min each, 45 min physical activity per day, 4–8 days (*M* = 8.4 days, *SD* = 2.52) Conducted by certified cognitive behavioural therapists and EMDR therapists	DITS-ID* (PTSD symptoms), SDQ* (emotional and behavioural problems)	Significant reduction in PTSD symptoms, emotional problems and behavioural problems from start to end of treatment. Results remained stable at 3-month follow-up.	Serious Risk (ROBINS-I)
Peters-Scheffer et al., [35]	Controlled Trial (CT), non-randomised pretest-posttest design CT	*N* = 34 IG: *n* = 12 CG: *n* = 22 Age: *M* = 53.50 months, *SD*: 5.25; CG: *M* = 52.95 months, *SD*: 11.14 ID: mixed severity (IQ not reported)	Low intensity behavioural treatment (average 6.5 h per week) supplementing preschool services, using techniques such as discrete trial training, structured play, and group activities, all based on principles of Applied Behavior Analysis (ABA); Conducted by trained clinicians	Mental Development Intex, (MDI)*; Vineland Adaptive Behavior Scales (VABS)*; Scale of Pervasive Developmental Disorder in Mentally Retarded Persons (PDD-MRS)*; Child Behavior Checklist (CBCL)*	Children receiving the behavioural treatment showed significantly higher developmental ages and gains in adaptive skills compared to the control group. No significant differences were found in the severity of autism symptoms between the groups.	Critical Risk (ROBINS-I)
Plant and Sanders, [36]	Randomised Controlled Trial (RCT), 3-arm (SSTP-S vs. SSTP-E vs. Waitlist), 1-year follow-up	*N* = 74 (families); Children < 6 years with developmental disability; ID: borderline to severe (4.6% borderline, 33.3% mild, 45.8% moderate, 16.7% severe; IQ not reported)	Stepping Stones Triple P (SSTP), a behavioural parent training program. Two active arms: Standard (SSTP-S, 10 sessions) and Enhanced (SSTP-E, 16 sessions)	DBC-D* (disruptive), ; DASS* (Depression, Anxiety, Stress)	Both intervention groups showed significant reductions in child problem behaviour compared to the waitlist. There were no significant differences between the two intervention types. No significant effect was found for parental distress (DASS).	(ROB2) High risk
Ratcliffe et al., [39]	Controlled Trial (CT), quasi-experimental, non-randomised, pre-post design	*N* = 75 Age range: 7–13 years (*M* = 9.3; *SD* = 1.43) IG: *n* = 43; 9 m/34f; age: *M* = 9.41 (*SD* = 1.48) CG: *n* = 32; 1 m/31f; age: *M* = 9.12 (*SD* = 1.36) ID: mild (IQ 50–70)	Emotion-Based Social Skills Training (EBSST) intervention, 16 sessions over three trimesters, 90 min per week; conducted by certified trainers	Emotional Development Questionnaire (EDQ*); Social Skills Improvement System (SSIS*); Developmental Behaviour Checklist (DBC)*	Emotional competence: statistically significant improvements in the TG compared to the CG, but not significant after Bonferroni correction. Effect size: Cohen’s d = 0.32. Social skills and mental health: No statistically significant differences between the groups after the intervention.	Moderate Risk (ROBINS-I)
Royston et al., [41]	RCT (single-blind)	*N* = 261 Age range: 2.5–5 years (*M* = 3.7 years, *SD* = 1.0) ID moderate to severe (ABAS 40–69; IQ not reported)	Stepping Stones Triple P (Level 4); 9-week program with group + individual components; delivered by trained staff	CBCL*, C-TRF*, Parenting Stress Index*, GHQ-12*, Caregiving Problem Checklist*	No significant effect in ITT; significant improvement in per-protocol and pre-COVID subgroup	Some Concerns (ROB 2)
Schuiringa et al., [44]	Randomised Controlled Trial (RCT), pre-post design, no follow-up	*N* = 169 IG: *n* = 97; 74% m; Age range: 9–16 (*M* = 13.27, *SD* = 1.88) CG: *n* = 72; 74% m; Age range: 9–16 (*M* = 13.19, *SD* = 1.79) ID: borderline to mild (IQ 55–85)	Stepping Stones Triple P (SSTP) parent training + Social Skills Training (SST) for children, 10 sessions of 90 min for parents, 12 sessions of 60 min for children; conducted by certified trainers	Measures (parenting, social cognitions): Alabama Parenting Questionnaire (APQ)* and Ghent Parental Behavior Scale (GPBS), Parenting Stress Index (PSI)*, Social Problem Solving Test for children with MBID (SPT-MID)* and Normative Beliefs About Aggression Scale (NOBAGS)*	Significant reduction in teacher-reported externalising behaviour (TRF) and increase in positive parenting and parent-child relationship in the intervention group (IG). Unexpected increase in aggressive social cognitions in IG. No significant effects on parent-reported externalising behaviour (CBCL, PDR) or negative parenting. Higher recovery rates based on teacher ratings at post-test in IG.	High Risk (ROB 2)
Stone-Heaberlin et al., [47]	Randomised Controlled Trial (RCT), double blind	*N* = 20 Age range: 6–17 years IQ: BEH group *M* = 50.9 (*SD* = 12.8) EDU group *M* = 45.8 (*SD* = 6.9 ID: moderate (IQ M = 48.4; Down syndrome)	Modified RUBI Parent Training (BEH) vs. psychoeducational control (EDU); 5 individual 1-hour sessions over 5–8 weeks; delivered by single trained facilitator	CBCL*, ABC*, BRIEF*, PSI*	Both conditions showed decreased externalising behaviour and hyperactivity; high feasibility and satisfaction; no group differences due to equal improvements	Some Concerns (ROB 2)
Te Brinke et al., [51]	Randomised Controlled Trial (RCT), micro-trial with two parallel conditions, multiple assessments across phases	*N* = 42 50% male; Age range 12–18 (*M* = 15.52, *SD* = 1.43), ID: borderline to mild (IQ 55–84)	“Think Cool, Act Cool” - training, consisting of cognitive module (Think Cool) and behavioural module (Act Cool), each with 5 individual sessions (in total 10 sessions, individual 60-min sessions) focused on emotion regulation skills, conducted by trained clinicians	Difficulties in Emotion Regulation Scale (DERS; self-report)*, (CBCL)*, (TRF)*; multi-informant, weekly measure of emotion regulation and externalising problem	Significant improvements in emotion regulation were observed when the cognitive module was administered before the behavioural module. Overall Improvement: Both sequences led to improvements in managing externalising behaviours, but the sequence starting with cognitive strategies was more effective.	High Risk (ROB 2)

ID = intellectual disability; RCT = randomised controlled trial; CT = controlled trial; IG = intervention group; CG = control group; CBT = cognitive behavioural therapy; PCIT = Parent-Child Interaction, PCI = Parenting Stress Index; Therapy; MST-ID = multisystemic therapy for intellectual disabilities; MMA = Mindfulness Martial Arts; RIT = Reciprocal Imitation Training; PMT = Parent Management Training; EMDR = Eye Movement Desensitization and Reprocessing; DBC = Developmental Behaviour Checklist - Disruptive subscale; DASS = Depression Anxiety Stress Scales; ABA = Applied Behavior Analysis; ABC = Aberrant Behavior Checklist; BRIEF = Behavior Rating Inventory of Executive Function; EBSST = Emotion-Based Social Skills Training; SSTP = Stepping Stones Triple P; SST = Social Skills Training; MBID = mild to borderline intellectual disability; PedsQL = Parenting Sense of Competence Scale; *M* = mean; *SD* = standard deviation; m/f = male/female; *n* = sample size; *N* = total number of participants; ROB 2 = Risk of Bias Tool 2; ROBINS-I = Risk of Bias in Non-randomised Studies of Interventions

*Outcome measures used for calculations in the meta-analysis are marked with an asterisk

ID severity: ID severity categories were reported as described by the original study authors. See moderator analysis section for classification criteria used in subgroup analyses

## Statistical analysis

For the calculation of effect sizes and subgroup analyses, the web-based version of Review Manager (RevMan Web), provided by the Cochrane Collaboration (https://revman.cochrane.org), was used. Effect sizes for behavioural problems and parental stress were calculated for each study. For studies with multiple active intervention groups but no inactive control group, we analysed each intervention arm separately as a pre-post comparison rather than comparing between intervention groups. The outcome measures selected from these studies are indicated in Table 1. Cohen’s *d* with a 95% confidence interval (CI) was calculated as the effect size. For controlled trials (CTs), the pooled standard deviation was calculated considering the group size [29]. For repeated measures within uncontrolled pre–post designs, effect sizes were calculated using the method proposed by Morris and DeShon [32], which includes a corrected standard deviation that accounts for the correlation between pre- and post-measurements. The magnitude of effect sizes was categorised according to Cohen [11]: small (0.20 to 0.49), medium (0.50 to 0.79), and large (0.80 and above). A *p*-value of less than 0.05 was considered statistically significant. All positive effect sizes were standardised to indicate better outcomes for the intervention group in RCTs or reductions in symptoms in CTs. Only post-treatment measures were included in the calculations due to varying follow-up periods across studies. Effects from RCTs and CTs were combined for each domain. The meta-analysis was performed using a random-effects model. Heterogeneity between studies was assessed using the *I²* statistic, representing the proportion of variance across studies that is due to heterogeneity rather than chance. *I²* values range from 0% to 100%. The results of the meta-analysis are presented in forest plots. In multi-arm trials with more than one relevant intervention group sharing a common control, intervention groups were combined to create a single comparison, following Cochrane guidelines. Specifically, in Plant and Sanders [36], the SSTP-Standard and SSTP-Enhanced conditions were pooled into one intervention arm contrasted against the waitlist control. Based on theoretical considerations and prior research suggesting differential treatment responsiveness, we pre-planned exploratory moderator analyses examining intervention type, format, and ID severity.

## Results

The initial database screening identified 2,010 records. Additional seven records were identified through other sources (*n* = 5 via handsearch, *n* = 2 via updated database searches), resulting in a total of 2,017 records prior to duplicate removal. After removing 382 duplicates, 1,635 records were screened based on their titles and abstracts, leading to the exclusion of 1,598 records. The most common reasons for exclusion were: studies focusing on autism spectrum disorder without co-occurring ID (35%); non-intervention studies such as prevalence estimates, diagnostic assessments, or qualitative research (18%); studies with irrelevant outcomes targeting cognitive, academic, or linguistic rather than psychological functioning (13%); wrong intervention type including pharmacological treatments, technology-based interventions, or complementary therapies such as music, animal-assisted, or exercise interventions (19%); studies focusing exclusively on adult populations (8%); and other reasons including reviews, study protocols without results, and irrelevant database hits (9%).

**Fig. 1:**
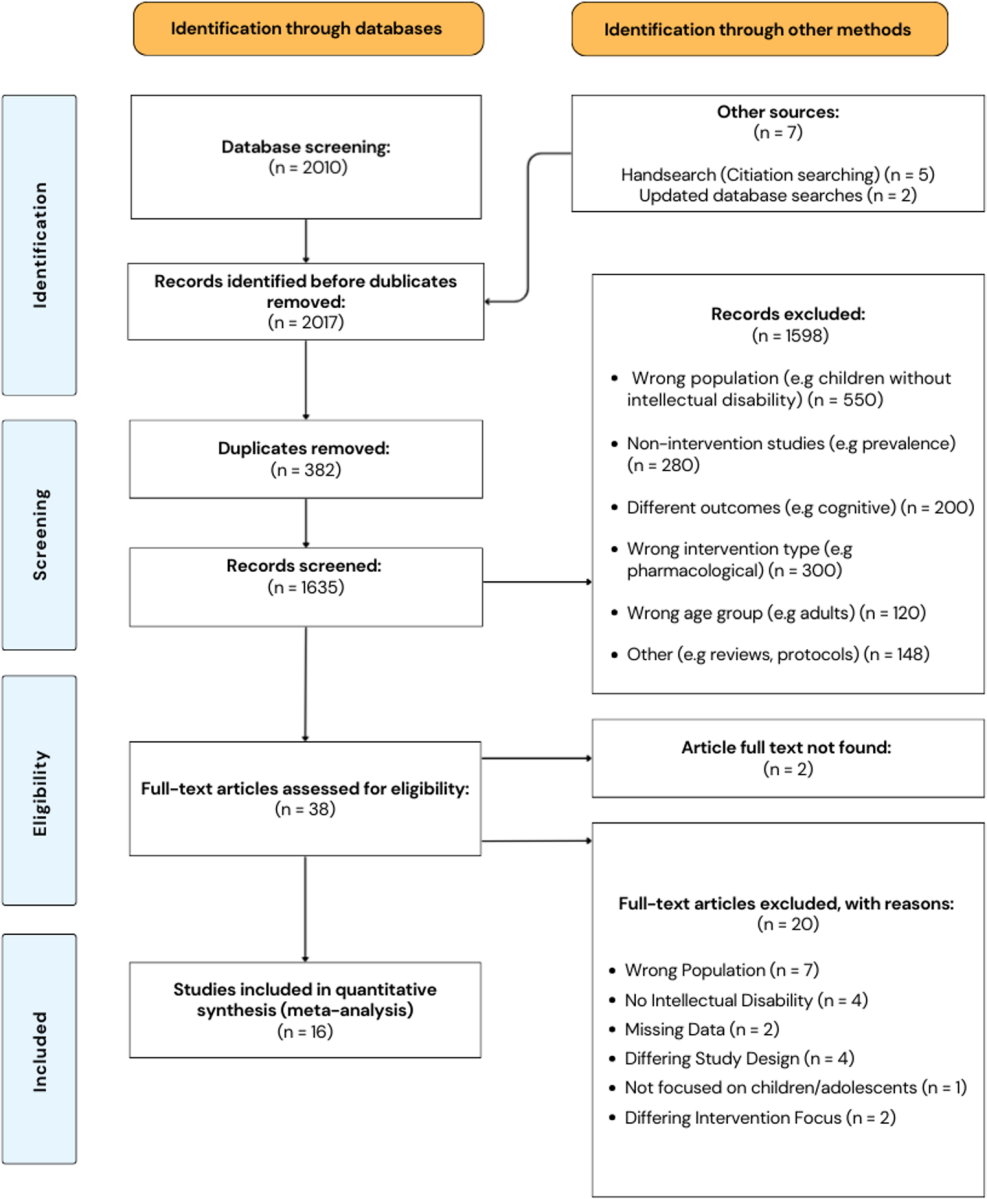
PRISMA flow chart of study selection. Note. PRISMA = Preferred Reporting Items for Systematic Reviews and Meta-Analyses

The full texts of the remaining 38 articles were then assessed for eligibility. Two of these articles could not be retrieved. Of the remaining 36 articles, a further 20 were excluded. The primary reasons for exclusion at this stage were: wrong population (e.g., studies focused on high-functioning autism without ID; *n* = 7), no participants with ID (*n* = 4), missing data that prevented analysis (*n* = 2), differing study design (*n* = 4), not focused on children or adolescents (*n* = 1), and a differing intervention focus (*n* = 2). This process resulted in a final sample of 16 studies that were included in the quantitative synthesis (meta-analysis). The complete study selection process is illustrated in the PRISMA flowchart (Fig. 1).

## Study characteristics

We included 16 studies published between 2007 and 2024, comprising a total of 1,084 participants aged 2.5 to 19 years. Ten studies were randomised controlled trials (RCTs), four were non-randomised controlled trials [7, 23, 35, 39], and two were uncontrolled pilot studies (Blakeley-Smith et al., 2020; Ooms-Evers et al., 2021). Overall, the average sample size per study was approximately 68 participants. The RCTs (*N* = 10) had sample sizes ranging from 20 to 261 participants. The most frequently applied interventions involved cognitive-behavioural approaches, parent management training (e.g., Parent-Child Interaction Therapy (PCIT); Parent Management Training (PMT); Stepping Stones Triple P; Incredible Years), social skills training, mindfulness-based interventions, emotion regulation training, and Applied Behaviour Analysis approaches. Two studies also incorporated trauma-focused interventions (EMDR in Ooms-Evers et al., 2021) and adapted multisystemic therapy (MST-ID in [7]). Five studies delivered their intervention in group settings ([23, 26, 39]; McIntyre, 2008; [20]), seven in individual sessions ([5, 24, 35, 47, 51]; Ooms-Evers et al., 2021; Blakeley-Smith et al., 2020), and two applied combined group plus individual formats [41, 44].

Fourteen studies included a control group. Four used a waitlist control [5, 23, 26, 36], while the others employed active or alternative comparisons (e.g., standard MST in [7]; psychoeducational control in [47]) or treatment-as-usual controls. Four studies ([5, 7]; Ooms-Evers et al., 2021; [36]) conducted follow-up assessments beyond immediate posttreatment (ranging from 3 months to 1 year). In terms of participant gender, the proportion of males ranged from 3% to 100% (where reported), with considerable variation across studies.

## Methodological quality

Based on RoB 2 for the RCTs, four studies ([26]; McIntyre, 2008; [41, 47]) received a rating of Some Concerns. Six RCTs [5, 20, 24, 36, 44, 51] had High Risk of Bias, primarily due to challenges in blinding and incomplete handling of missing data.

The non-randomised studies, evaluated using ROBINS-I, showed varying risk levels: one study [39] was rated as Moderate Risk, one study (Ooms-Evers et al., 2021) as Serious Risk, while four studies (Blakeley-Smith et al., 2020; [7, 23, 35]) were classified as Critical Risk, often because of confounding factors, baseline imbalances that were not adjusted statistically, or lack of control groups.

## RCTs and CTs

### Behavioural disorders

**Fig. 2:**
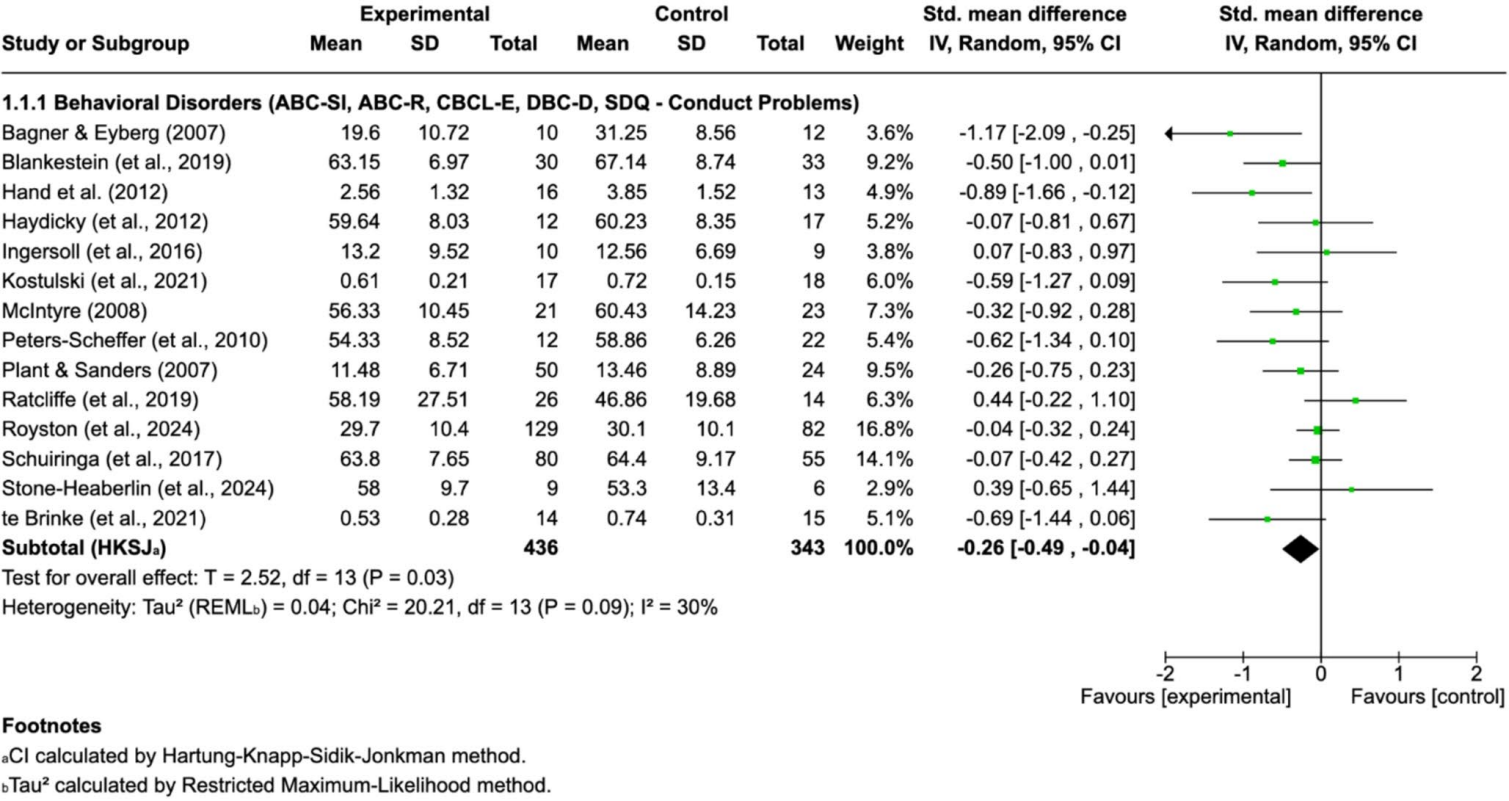
Forest-plot of controlled studies behavioural disorders (RCTs and CTs)

The primary analysis of 14 controlled trials (*N* = 779) revealed a small but statistically significant effect on behavioural disorders (*SMD* = −0.26, 95% CI [−0.49, −0.04], *p* = .03). The analysis indicated low to moderate heterogeneity (*I²* = 30%) that was not statistically significant (*p* = .09), suggesting a relatively consistent effect across the included studies. The overall effect was calculated using the Hartung-Knapp-Sidik-Jonkman method, which provides more robust estimates for meta-analyses with a limited number of studies (see Fig. 2).

#### Moderator analysis

Although the overall test for heterogeneity was not statistically significant (*I²* = 30%, *p* = .09), we conducted three pre-planned exploratory moderator analyses based on a-priori hypotheses about factors likely to influence intervention efficacy. These examined the influence of intervention type, format, and participant ID severity.

#### Intervention type: parent- vs. child-focused

First, to explore the sources of heterogeneity, a subgroup analysis was conducted based on the primary intervention type. The analysis compared parent-mediated interventions (*k* = 8) with child-focused interventions (*k* = 6). The test for subgroup differences revealed no statistically significant difference between the two approaches (*Q*(1) = 0.02, *p* = .90). The pooled effect for parent-mediated interventions (*SMD* = −0.27, 95% CI [−0.60, 0.05]) was nearly identical to the effect for child-focused interventions (*SMD* = −0.25, 95% CI [−0.72, 0.23]). Thus, intervention type did not appear to be a significant source of heterogeneity in this analysis.

#### Intervention format: individual vs. group

In contrast, the intervention format emerged as a significant moderator of treatment efficacy. Here the analysis revealed a statistically significant difference between the subgroups (*Q*(2) = 8.71, *p* = .01). Interventions delivered in an individual format (*k* = 7) showed a medium, statistically significant effect (*SMD* = −0.46, 95% CI [−0.82, −0.10]). In contrast, interventions delivered in a group format (*k* = 5; *SMD* = −0.27) or a combined format (*k* = 2; *SMD* = −0.05) did not yield statistically significant effects. These findings suggest that the intervention format is a significant moderator, with individual or dyadic approaches appearing substantially more effective than group-based models for treating behavioural disorders in this population.

#### Severity of intellectual disability

Finally, a subgroup analysis was conducted to test the hypothesis that the severity of ID would moderate the treatment effect. Based on the participant characteristics reported in each study, studies were categorised into two subgroups: (1) *Mild/Borderline ID* (k = 6), including studies where samples consisted of participants with mild or borderline intellectual disability (IQ approximately 55–85), and (2) *Mixed/Severe ID* (k = 8), including studies where samples comprised participants across multiple severity levels or included individuals with moderate to severe ID (IQ < 55). The analysis revealed no statistically significant difference between the two subgroups (*Q*(1) = 0.02, *p* = .90). The pooled effect for the Mixed/Severe ID group (*SMD* = −0.28, 95% CI [−0.61, 0.04]) was nearly identical to that of the Mild/Borderline ID group (*SMD* = −0.25, 95% CI [−0.73, 0.22]). Thus, ID severity, as categorised in this analysis, did not appear to explain the variability in treatment efficacy.

#### Analysis of publication bias

**Fig. 3:**
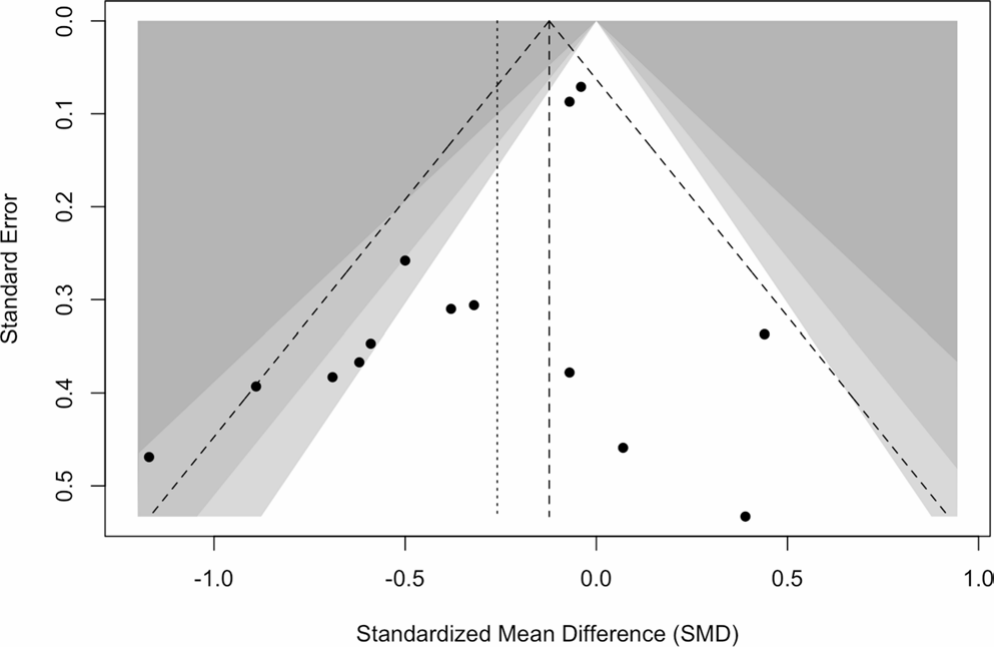
Contour-enhanced funnel plot of included studies assessing behavioural disorders

Publication bias was assessed using a contour-enhanced funnel plot and Egger’s linear regression test. Visual inspection of the plot revealed asymmetry, with an absence of smaller studies reporting non-significant or positive effects. This visual impression was confirmed by a statistically significant Egger’s test (*t*(12) = −2.19, *p* = .049), suggesting the summary effect may be an overestimation due to the potential underrepresentation of studies with null or unfavourable results, displayed in Fig. 3.

## Parental stress

**Fig. 4:**
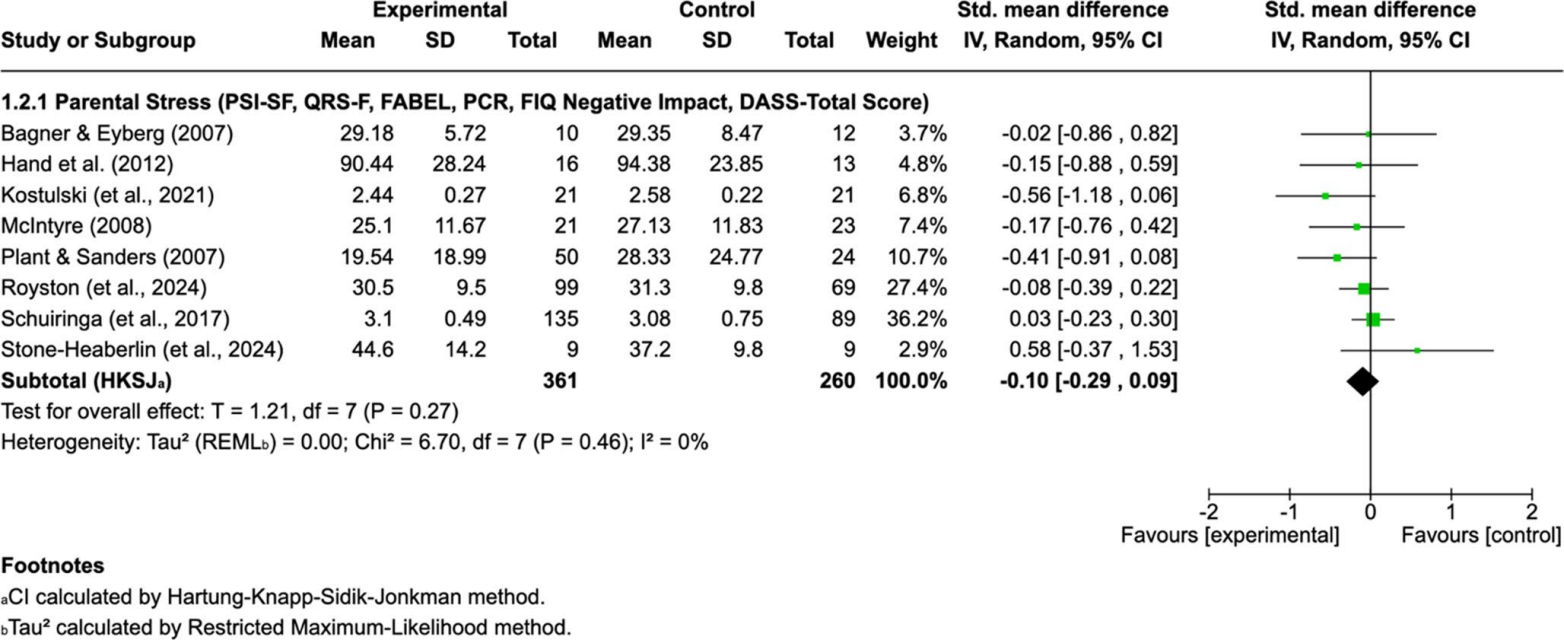
Forest-plot of controlled studies of parental stress (RCTs and CTs)

The analysis of eight controlled trials (*N* = 621) investigating the effects on parental stress demonstrated a small, non-significant overall effect (*SMD* = −0.10, 95% CI [−0.29, 0.09], *p* = .27). A complete absence of heterogeneity was observed (*I²* = 0%), indicating that this null finding was highly consistent across all included studies (see Fig. 4).

## Uncontrolled trials

### Behavioural disorders

**Fig. 5:**
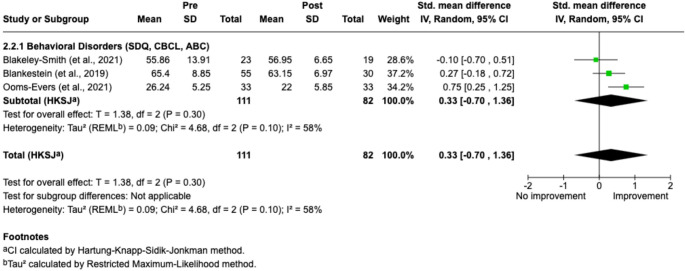
Forest-plot of uncontrolled studies of behavioural disorders

Three uncontrolled studies (*N* = 111 pre-intervention, *N* = 82 post-intervention) addressing behavioural disorders reported a moderate, non-significant overall effect (*SMD* = 0.33, 95% CI [−0.70, 1.36], *p* = .30) with substantial heterogeneity (*I²* = 58%, *p* = .10). The interventions showed considerable variation in their efficacy for improving behavioural problems. Blakeley-Smith et al. (2021) using adapted cognitive-behavioural therapy demonstrated a small negative effect (*SMD* = −0.10, 95% CI [−0.70, 0.51]), Blankenstein et al. (2019) employing MST-ID showed a small positive effect (*SMD* = 0.27, 95% CI [−0.18, 0.72]), while Ooms-Evers et al. (2021) utilising trauma exposure and EMDR therapy demonstrated a large significant improvement (*SMD* = 0.75, 95% CI [0.25, 1.25]). The substantial heterogeneity likely reflects differences in intervention approaches, target populations, and the specific behavioural problems addressed across studies. A visual overview of the data is provided in Fig. 5.

## Anxiety

**Fig. 6:**
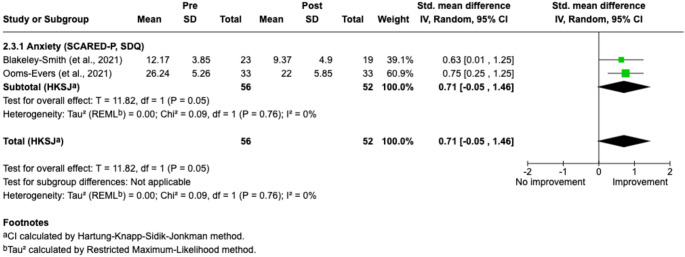
Forest-plot of uncontrolled studies on anxiety

Two uncontrolled trials (*N* = 56 pre-intervention, *N* = 52 post-intervention) examining anxiety-focused interventions produced a large overall effect (*SMD* = 0.71, 95% CI [−0.05, 1.46], *p* = .05), with no heterogeneity (*I²* = 0%, *p* = .76). Blakeley-Smith et al. (2021), employing an adapted CBT protocol for co-occurring ASD and anxiety, demonstrated a moderate to large effect (*SMD* = 0.63, 95% CI [0.01, 1.25]), while Ooms-Evers et al. (2021), using trauma exposure and EMDR therapy, showed a large effect (*SMD* = 0.75, 95% CI [0.25, 1.25]). Both studies showed statistically significant individual improvements in anxiety symptoms, with the overall effect approaching significance. As can be seen in Fig. 6.

## Depression

**Fig. 7:**
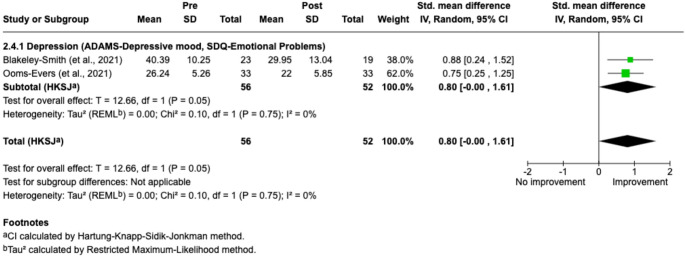
Forest-plot of uncontrolled studies on depression

The analysis of two uncontrolled studies (*N* = 56 pre-intervention, *N* = 52 post-intervention) targeting depressive symptoms demonstrated a large overall effect (*SMD* = 0.80, 95% CI [−0.00, 1.61], *p* = .05) with no heterogeneity (*I²* = 0%, *p* = .75). Both Blakeley-Smith et al. (2021; *SMD* = 0.88, 95% CI [0.24, 1.52]) and Ooms-Evers et al. (2021; *SMD* = 0.75, 95% CI [0.25, 1.25]) showed statistically significant improvements in depressive symptoms, displayed in Fig. 7. The consistency across these studies, despite differences in their primary therapeutic approaches (adapted CBT versus trauma-focused therapy), suggests that structured therapeutic interventions may have beneficial effects on depressive symptoms even when depression is not the primary treatment target.

## Discussion

This meta-analysis of 16 studies provides a comprehensive overview of psychotherapeutic interventions for youth with ID, offering a nuanced picture of the current evidence. The analysis establishes a small, significant therapeutic effect for behavioural disorders, a finding which appears to be primarily driven by interventions delivered in an individual format. This result should be interpreted with consideration of potential publication bias. In addition, the analysis found no significant overall effect on parental stress and highlights a clear need for more robust, controlled research for internalising disorders.

### Effects on behavioural disorders

Our meta‑analysis demonstrated that psychotherapeutic interventions can yield modest reductions in behavioural problems in youth with ID. Importantly, subgroup analyses indicated that the severity of ID did not moderate treatment effects, children with severe disabilities benefited comparably to those with mild or borderline impairments when interventions were tailored using simplified language, visual supports and active involvement of caregivers. This finding counters the assumption that treatment efficacy diminishes as cognitive impairment increases. It accords with an earlier meta-analysis of interventions for challenging behaviour in adults across the full range of intellectual disability, which found no significant moderation by severity or age [22].

Koslowski et al., [27] and Vereenooghe and Langdon [53], likewise observe substantial reductions in aggressive or disruptive behaviour among adults with mild to moderate ID. The comparatively small effect in our review ( = −0.26) likely reflects developmental factors: children with ID have less developed self‑regulation skills, higher rates of comorbid neurodevelopmental disorders and rely more on caregivers to implement strategies. Evidence from adult populations further supports a developmental gradient: while Heyvaert et al., [22] and Graser et al., [18] found medium to large effects in adults, effect sizes in children remain smaller, suggesting that therapeutic interventions may become more effective with increasing cognitive maturity.

Our analyses also highlight that the intervention format is more critical than severity level of cognitive impairment. Only individual or dyadic formats produced significant improvements, whereas group‑based programmes did not. Standardised group curricula such as Triple P, which have demonstrated effectiveness in families of typically developing children, may not provide enough individualised support for the heterogeneous needs of children with ID. Among the seven individually delivered interventions, five were parent-mediated [5, 7, 24, 36, 47] and two were child-focused [35, 51]. Therefore, the superiority of individual formats cannot be attributed to intervention type alone. The benefit of individual delivery may instead reflect the need for tailored adaptations given the heterogeneous cognitive, communicative, and behavioural profiles of children with ID. This interpretation is consistent with findings from the broader parent training literature. In a meta-analysis of 63 studies, Lundahl et al., [31] found that individually delivered parent training produced significantly greater improvements in child behaviour than group delivery. Notably, this advantage was particularly pronounced for families facing greater challenges, suggesting that individualisation is especially important when treatment needs are complex. Hybrid models combining small group sessions with individual coaching may warrant exploration.

A useful benchmark is provided by the recent meta-analysis of Kühl et al., [28], which, based on seven studies and a multilevel approach, reported a larger effect (g = 0.49). Their narrower focus on mild ID and highly standardised programmes contrasts with our broader evidence base of 13 studies spanning the full severity range. While Kühl et al., [28] delineate the upper-bound potential of interventions under favourable conditions, our findings offer a more conservative and ecologically valid estimate, highlighting the greater efficacy of individually delivered formats and the need for adaptive designs across heterogeneous clinical populations.

In addition uncontrolled trials on behavioural disorders complement these findings but underline their limitations. The pooled effect was moderate but non-significant, with results ranging from negative (adapted CBT; Blakeley-Smith et al., 2021) to strongly positive (trauma-focused EMDR; Ooms-Evers et al., 2021). This heterogeneity reflects the diversity of approaches and populations, and while such studies cannot provide reliable evidence, they generate hypotheses and suggest that targeted, trauma-focused treatments may hold promise for specific subgroups.

### Evidence for internalising disorders

For anxiety and depression uncontrolled pilot studies suggested large effects. Yet, with only two studies per outcome and no control groups, these findings lack evidential robustness. Phenomena such as regression to the mean, placebo effects, or natural symptom fluctuations cannot be ruled out, meaning these results do not provide reliable evidence for the efficacy of interventions.

Further, the lack of robust trials in pediatric populations is striking. While Graser et al., [18] included eight controlled studies on depression in adults with ID, and Reynolds et al., [40] synthesised 55 RCTs in typically developing youth, no controlled trials were identified in our review. This confirms earlier observations by Cameron et al., [9] and underscores a critical research gap: internalising disorders in children with ID remain profoundly neglected, leaving clinical practice without an evidence-based foundation.

### Effects on parental stress

The null findings for parental stress is arguably one of the most critical takeaways of this analysis. This stands in contrast to recent findings in children with mild or borderline intellectual disability, where interventions yielded a significant, though small, reduction in parental stress [28]. Ranta et al. [38] reviewed 21 studies of parent‑focused interventions and reported moderate to large improvements in parental well‑being, although most samples involved families of children with mild or moderate disability and were subject to high risk of bias. This suggests that the benefits of child-focused interventions on parental stress may be confined to families of children with fewer functional limitations, rather than extending to the broader ID population.

Furthermore, parental stress may be influenced by factors extending beyond the child’s behavioural presentation, including financial concerns, social support networks, respite care availability, and long-term care planning considerations that therapeutic interventions may not directly address. Research has identified that approximately 31% of parents of children with intellectual and developmental disabilities reach clinical cutoffs for moderate depression and anxiety, with stress encompassing four major dimensions: parental gender differences, diagnosis-related coping issues, socioeconomic characteristics, and social isolation [43]. Parental stress could therefore be a deeply rooted issue that often persists for years and may require more intensive, long-term support.

### Implications for clinical practice and research

For clinical practice, this work provides several specific, evidence-based messages. First, while psychotherapeutic interventions for behavioural disorders are effective, our findings strongly suggest that the choice of intervention format is critical. Within these effective formats, systematic adaptation is key. This involves not only simplifying content through short, concrete sentences and pictorial supports (e.g., pictograms, social stories), but also adapting delivery methods to emphasise behavioural rehearsal techniques such as structured role-play and in-session modelling. Equally important is the systematic involvement of parents as active co-therapists, for example by training them to use daily homework tasks, reinforcement schedules, and emotion-coaching strategies to implement skills consistently at home [50, 53].

Second, the absence of effects on parental stress shows that caregiver burden must be treated as an independent treatment goal. Child-focused approaches alone are insufficient, and specific modules addressing parental stressors are required. Third, given the extremely limited evidence for anxiety and depression, interventions in this domain should be treated as individualised therapeutic trials. However, preliminary signals from uncontrolled data suggest that for children whose issues may be trauma-related, specific trauma-focused interventions could be particularly promising.

For research, the most urgent priority is to plan and administer methodologically rigorous, large-scale randomised controlled trials (RCTs) to overcome the current evidence base marked by bias and low statistical power. Future studies should go beyond efficacy testing by incorporating planned moderator and mediator analyses to clarify what works for whom and under which conditions. Particular attention should be given to the therapeutic alliance, child cognitive level, and parental factors such as stress or mental health. Finally, progress in this field depends on developing and validating instruments that can reliably measure symptoms and outcomes across the spectrum of intellectual disability. Without such tools, study findings will remain difficult to interpret.

### Strengths and limitations

This meta-analysis has several noteworthy strengths. It represents the most comprehensive synthesis of psychotherapeutic interventions for children with ID to date, providing a broader evidence base than prior work. By systematically differentiating between outcomes for child behaviour, parental stress, and internalising symptoms, the analysis offers a nuanced understanding of where current approaches are most effective and where critical evidence gaps remain. Furthermore, the review adhered to rigorous methodological procedures for study identification, risk of bias assessment, and statistical analysis, ensuring transparency and reproducibility.

At the same time, the findings must be interpreted in light of several limitations, the primary being the significant evidence of publication bias, as well as concerns regarding the methodological quality of the included studies. With a substantial portion rated at a high risk of bias due to challenges in blinding and handling of missing data, our main finding for behavioural disorders should be interpreted with caution as it may be an overestimation of the true effect. Furthermore, the limited number of studies per outcome restricted statistical power. This not only tempers confidence in the main findings but also limits the ability to investigate the moderate heterogeneity beyond exploratory moderator analyses. Finally, the complete absence of controlled trials for internalising disorders is a major limitation inherited from the primary literature, preventing any firm conclusions about treatment efficacy for anxiety and depression in this population.

## Conclusion

This meta-analysis demonstrates that psychotherapy can reduce behavioural problems in children and adolescents with intellectual disability, independent of impairment severity, although effect sizes are smaller than those observed in typically developing youth and in adults with ID. Its contribution lies in providing the first comprehensive and clinically realistic benchmark of treatment effects in this population, consolidating a fragmented literature into the largest synthesis to date. The real challenge now is to move beyond scattered, small-scale trials and to generate rigorous, large RCTs that integrate family-focused modules and address internalising symptoms. Only then can psychotherapeutic care for this underserved group progress from fragile promise to an established standard of evidence-based practice.

## Data Availability

Data and analysis materials are available on the Open Science Framework (OSF): 10.17605/OSF.IO/V6ETU.
